# Clinically unsuspected orthopedic implants during *S. aureus* bacteremia do not require additional diagnostic work-up

**DOI:** 10.1007/s15010-022-01913-9

**Published:** 2022-09-08

**Authors:** Ilse J. E. Kouijzer, L. T. D. Speijker, E. H. J. G. Aarntzen, W. H. C. Rijnen, M. P. Somford, I. Maat, M. P. A. van Meer, J. Ten Oever, E. H. Gisolf

**Affiliations:** 1 Department of Internal Medicine and Radboud Center for Infectious Diseases, Radboud University Medical Center, P.O. Box 9101, 6500 HB Nijmegen, the Netherlands; 2 Department of Medical Imaging, Radboud University Medical Center, Nijmegen, the Netherlands; 3 Department of Orthopaedics, Radboud University Medical Center, Nijmegen, the Netherlands; 4 Department of Orthopaedics, Rijnstate Hospital, Arnhem, the Netherlands; 5 Department of Medical Microbiology and Radboud Center for Infectious Diseases, Radboud University Medical Center, Nijmegen, the Netherlands; 6 Department of Medical Microbiology and Immunology, Rijnstate Hospital, Arnhem, the Netherlands; 7 Department of Internal Medicine, Rijnstate Hospital, Arnhem, the Netherlands

**Keywords:** Orthopedic implants, *Staphylococcus aureus* bacteremia, FDG-PET/CT, Diagnostics

## Abstract

**Purpose:**

To assess the likelihood of occult infection in patients with clinically unsuspected orthopedic implants during *Staphylococcus aureus* bacteremia (SAB).

**Methods:**

In a retrospective study in two Dutch hospitals, we included all patients with SAB between 2013 and 2020 with one or more orthopedic implants in whom [^18^F]FDG-PET/CT was performed. The primary outcome was the percentage of patients who had an orthopedic implant-related infection by *S. aureus*. We also compared clinical parameters in patients with clinically suspected and unsuspected implants.

**Results:**

Fifty-five of 191 (29%) orthopedic implants in 118 SAB patients included had clinical signs of infection. Of all 136 unsuspected implants, 5 (3%, all arthroplasties), showed increased [^18^F]FDG uptake around the prosthesis on [^18^F]FDG-PET/CT. The clinical course of these patients without clinically overt infection or relapse of bacteremia during follow-up of a median of 48 months (range 0–48), however, argued against prosthetic joint infection.

**Conclusion:**

Although orthopedic implants are evidently a risk factor for metastatic infection during SAB, the absence of clinical symptoms obviate the need of additional investigations or prolonged antibiotic treatment.

## Introduction

Orthopedic implant-related infection is a notorious complication of *Staphylococcus aureus* bacteremia (SAB) [1]. In clinical practice, the presence of orthopedic implants in patients with SAB, therefore, commonly leads to additional imaging or the consideration to aspirate a prosthetic joint [2, 3]. Current guidelines classify SAB as complicated if the patient has prosthetic material and, therefore, recommend prolonged treatment [4]. However, such an approach is questionable in orthopedic implants clinically unsuspected for infection. A recent study observed a low risk of a prosthetic joint infection (PJI) if asymptomatic during a concomitant hematogenous PJI of another joint [5]. In a small previous study, we found that 2-[^18^F]fluoro-2-deoxy-d-glucose positron emission tomography with combined computed tomography ([^18^F]FDG-PET/CT) showed no infection in the seven SAB patients with clinically unsuspected orthopedic implants [6]. In this study, we assess the likelihood of occult infection in patients with clinically unsuspected orthopedic implants using [^18^F]FDG-PET/CT in a large multi-center cohort of SAB patients.

## Methods

In this retrospective multi-center cohort study, all adults with SAB between 2013 and 2020 were included if (i) they had one or more orthopedic implants and (ii) [^18^F]FDG-PET/CT was performed. Orthopedic implants included prosthetic joints and osteosynthesis material such as plates, rods, and intramedullary nails. Patients who died within 72 h after the first positive blood culture with *S. aureus* were excluded. This study was performed in two Dutch hospitals: one university hospital and one large teaching hospital. The regional institutional ethics committee approved this study and the requirement to obtain informed consent was waived (nr 2020-7104). We manually retrieved the clinical data from the electronic medical charts. [^18^F]FDG-PET/CT scans were not revised. The primary outcome measure was the percentage of patients who had an orthopedic implant-related infection with *S. aureus*. We also compared clinical parameters in patients with clinically suspected and unsuspected implants.

Definitions for proven, probable, unlikely, and rejected diagnosis of orthopedic implant-related infection, including compatible clinical signs of a clinically suspected implant-related infection, are shown in Table 1. An orthopedic implant-related infection within 30 days after surgery was considered a postoperative wound infection. Cure was defined as the patient being alive without signs and symptoms of infection 3 months after discontinuation of antibiotic treatment. A new episode of SAB within this time frame was considered a relapse.

**Table 1:** Definitions for orthopedic implant-related infection

	Definition
Proven infection	A positive culture with *S. aureus* from the implant site [14] with compatible clinical signs such as pain, redness, swelling, impaired function
Probable infection	Above clinical signs of infection and increased [^18^F]FDG uptake of the orthopedic implant
Unlikely infection	Absence of above clinical signs but increased [^18^F]FDG uptake of the orthopedic implant
Rejected infection	No increased [^18^F]FDG uptake of the orthopedic implant

SPSS (Version 25.0; SPSS, Inc.) was used for data analysis. The unpaired student’s *t-*test, Mann–Whitney *U* test, chi-square test or Fisher’s exact test were used to compare groups, where appropriate. Statistical significance was defined as a two-sided *P* value of < 0.05.

## Results

One hundred and eighteen SAB patients with a total of 191 orthopedic implants were included (Table 2). Of these 118 patients, 48 (41%) had one or more clinically suspected orthopedic implants and, therefore, a proven (73%) or probable (27%) implant-related infection. Of the 191 orthopedic implants, 138 were prosthetic joints and 53 osteosynthesis material. Fifty-five implants (29%) had clinical signs of infection: 33 PJI (60%; proven 25; probable 8) and 22 osteosynthesis infections (OI) (40%; proven 12; probable 10). Four of 33 PJI (12%) and 8 of 22 OI (36%) were postsurgical deep wound infections.

**Table 2:** Baseline characteristics and outcome of all patients

	SAB patients with clinically orthopedic implant-related infection, *n* = 48	SAB patients without clinically orthopedic implant-related infection, *n* = 70	*P* value
Male (%)	29 (60)	32 (46)	0.136
Age (mean, IQR)	65.4 (21)	74.6 (14)	0.003
Community-acquisition (%)	39 (81)	46 (86)	0.180
Persistent fever > 72 h (%)	22 (46)	16 (23)	0.012
Persistent blood cultures > 48 h (%)	21 (44)	43 (61)	0.058
Delay between onset and start of treatment > 48 h (%)	19 (40)	19 (27)	0.155
Charlson Comorbidity Index* (mean, IQR)	3.7 (3)	6.9 (4)	< 0.001
Diabetes mellitus (%)	11 (23)	24 (34)	0.938
Malignancy (%)	6 (13)	12 (17)	0.301
Renal failure (%)	2 (4)	14 (20)	0.014
Heart valve prosthesis	0	0	
Vascular graft prosthesis	0	0	
Pacemaker/ICD	0	0	
Overall number of involved joints**	55	136	
Location of prosthetic joint			
Hip	13	55	
Knee	17	40	
Shoulder	2	9	
Elbow	1	1	
Location of osteosynthesis material**			
Femur	6	11	
Pelvis	3	0	
Tibia	3	2	
Ankle	3	6	
Foot	0	4	
Vertebra	7	1	
Arm	0	2	
Hand	0	3	
Jaw	0	2	
Treatment duration, weeks (mean, IQR)	14.3 (12)	7.1 (4)	0.003
Mortality***	9 (19)	19 (27)	0.292
Relapse***	1 (2)	1 (1)	0.787

*Comorbidity was reported using the Charlson comorbidity score [15]

**Total of 191 orthopedic implants in 118 patients

***Follow-up was missing in 4 patients

Of all 136 orthopedic implants without clinical signs of infection, [^18^F]FDG-PET/CT showed increased [^18^F]FDG uptake in 5 prosthetic joints (5%) (4 patients). Of these 5 prosthetic joints, 3 were considered due to polyethylene wear (2 patients) [7]. In one patient with rheumatoid arthritis treated with etanercept and prednisone, [^18^F]FDG uptake was present around the clinically silent right hip prosthesis. A follow-up [^18^F]FDG-PET/CT after 3 months of antibiotic treatment showed unchanged [^18^F]FDG uptake around the prosthesis. No relapse occurred after discontinuation of antibiotics. [^18^F]FDG-PET/CT showed increased [^18^F]FDG uptake in the shoulder prosthesis of another patient who received chronic suppressive treatment for an infection of this prosthesis by *Candida albicans*. She had a new septic arthritis of the left shoulder without prosthesis but unchanged complaints on the right side. The left shoulder was drained. She died one month after SAB diagnosis due to frailty and heart failure without signs of a relapse of SAB. In these 4 patients with an unlikely diagnosis of PJI, median follow-up without any relapse was 48 months (range 0–48). In 2 of the 131 implants where infection was rejected, the patient had pain at the implant site. In these 2 patients, [^18^F]FDG-PET/CT did not show uptake around the implants.

## Discussion

This multi-center study in 118 patients with 191 orthopedic implants showed that 29% of them became infected during SAB, but that the risk of silent implant-related infection—as assessed by [^18^F]FDG-PET/CT—was negligible if clinical signs of infection were absent.

This larger study confirms the suggestion we found in a small subgroup of patients with orthopedic implants in our study where we refined the role of [^18^F]FDG-PET/CT in SAB [6]. Likewise, our findings are consistent with a recent study in the presence of asymptomatic PJI during bacteremia. In that study, however, only over one-third of the bacteremia episodes was due to *S. aureus* [5]. Tande et al. found 4 PJI in 50 SAB patients with 71 asymptomatic arthroplasties who had an infection of another arthroplasty [8]. These infections occurred between 174 and 670 days after SAB clearance, making the relationship to the initial bacteremia questionable. In another prospective cohort study of 143 SAB patients, no PJI developed during follow-up in the patients without apparent PJI at onset of bacteremia [9].

A strength of the current study is that in all patients an [^18^F]FDG-PET/CT was performed at the time of bacteremia, which was not standard procedure in other studies. [^18^F]FDG-PET/CT is a very sensitive nuclear imaging technique [10] that is increasingly used to detect metastatic infections in patients with SAB [3] 11. Although [^18^F]FDG is a very sensitive tracer for diagnosis of infection, discrimination between infection and inflammation may be difficult [12]. [^18^F]FDG uptake can also occur due to postsurgical inflammation, development of wear particles, and subtle aseptic loosening [13]. Correspondingly, in our study there were 5 patients without symptoms in whom [^18^F]FDG uptake was indeed not due to infection.

The results of this study contribute to the individualized diagnostic work-up in SAB patients. The presence of clinically silent orthopedic implants in patients with SAB should not lead to additional imaging such as [^18^F]FDG-PET/CT, and/or joint aspirations. Also, clinically unsuspected orthopedic implants should not affect treatment duration in patients with SAB. The latter is supported by a similar mortality in low-risk SAB patients with clinically unsuspected implants compared to patients without implants, while treatment duration was equally short [14].

Limitations of this study are the retrospective design and the inclusion of only patients who underwent [^18^F]FDG-PET/CT which may have led to selection bias. Although this impact of the latter is likely to be limited, because in our clinics [^18^F]FDG-PET/CT is mainly omitted in patients at low risk of dissemination or with already proven metastatic infection [3, 6]. As scans were not revised, interpretation of increased [^18^F]FDG uptake in clinically silent orthopedic implants may have been inconsistent, however, follow-up in these patients made the diagnosis of orthopedic implant-related infection unlikely.

In conclusion, although orthopedic implants are a clear risk factor for metastatic infection during SAB, the absence of clinical symptoms obviates the need of additional investigations or prolonged antibiotic treatment.

## Data Availability

To be requested from the authors.
